# Database of fish fauna collected from river channels on alluvial fans in the Kofu Basin, central Japan

**DOI:** 10.3897/BDJ.13.e159810

**Published:** 2025-10-07

**Authors:** Rei Itsukushima, Kazuaki Ohtsuki, Kota Tawa, Tatsuro Sato, Kazuki Karasawa

**Affiliations:** 1 Kyushu University, Fukuoka, Japan Kyushu University Fukuoka Japan; 2 Kyushu Institute of Technology, Kitakyushu, Japan Kyushu Institute of Technology Kitakyushu Japan; 3 University of Yamanashi, Kofu, Japan University of Yamanashi Kofu Japan; 4 National Institute for Environmental Studies, Tsukuba, Japan National Institute for Environmental Studies Tsukuba Japan

**Keywords:** anthropogenic impact, ichthyofauna, watershed geology, natural disasters

## Abstract

**Background:**

Alluvial fans, located near mountainous regions, are characterised by substantial sediment supply, large variations in flow and significant disturbances. The river channels on alluvial fans support a distinct biological community compared to those on alluvial plains. In Japan, gravel extraction and riverbed dredging to maintain flood discharge capacity were frequently carried out during the rapid economic growth period of the 1960s, leading to significant human impact on these rivers. As a result of frequent disturbances, the fish communities in these rivers tend to be less diverse. Furthermore, research on river channels in alluvial fans is limited compared to that on alluvial plains, resulting in a lack of detailed information on these ecosystems. Consequently, efforts for ecosystem conservation and restoration in these environments are insufficient.

**New information:**

A fish survey was conducted at a total of 26 sites across four river channels on alluvial fans, each with distinct geological characteristics. The results revealed the presence of 2,265 individuals belonging to 15 species and one genus (only the genus could be identified) within seven families. Amongst the confirmed species were *Misgurnus anguillicaudatus* (Cantor, 1842), *Oncorhynchus masou ishikawae* (Jordan and McGregor, 1925) and *Cottus pollux* Günther, 1873, all of which are listed in the local Red Data Book, highlighting the presence of rare species even in river channels on alluvial fans impacted by human activity. Furthermore, non-native species were found only at a single site in the upstream section, where four individuals of *Oncorhynchus mykiss* (Walbaum, 1792) were collected. The survey indicated that species diversity and fish species composition varied across the different rivers, suggesting that various factors, such as watershed geology, geomorphological processes and anthropogenic influences, contribute to differences in fish community structure.

## Introduction

Alluvial fans are the most widespread depositional landforms, occurring along the margins of long-standing highland areas or around actively subsiding continental basins, spanning a wide range of structural and climatic environments (Ventra and Clarke 2018). Rivers flowing through alluvial fans exhibit unique channel characteristics, such as diverse channel migrations and braided river systems influenced by sediment deposition, making them distinct from other river systems (Blair and Mcpherson 1994, Liu et al. 2024). The river channels on alluvial fans, located near mountainous regions, are characterised by substantial sediment supply, large fluctuations in flow and significant disturbances, which contribute to the formation of a distinct biological community compared to rivers in the alluvial plains. In New Zealand, the genetic structure of *Galaxias vulgaris* Stokell, 1949, differs between alluvial fan rivers and other river systems. In particular, in the braided river channels of alluvial fans, the genetic divergence is shallower due to the instability of the channels (Wallis et al. 2008). Additionally, even within the alluvial fan, the fish communities in the main river and the floodplain are distinct, with the spring-fed areas at the fan's edge acting as hotspots. It has been noted that the diversity of the main river is relatively low (Hirai 1974).

In alluvial fans, the large amount of sediment supplied from mountainous areas leads to the frequent deposition of sediment within the riverbed (Ishikawa 1986) and riverbed dredging is regularly carried out to maintain the river’s flood discharge capacity (Ikeda et al. 2017). Additionally, during Japan's rapid economic growth in the 1960s, there was a significant demand for gravel and large-scale sediment extraction was conducted in river channels on alluvial fans (Kayaba 2000, Watanabe et al. 2006). Such dredging activities, which involve alterations to the channel morphology, typically lead to habitat modifications, resulting in a decline in biodiversity. This has been reported in both alluvial fan rivers and other river systems (Kondolf 1994, Ellery and McCarthy 2002). Although river channels on alluvial fans are strongly influenced by both natural disturbances and anthropogenic impacts, the fish communities in these rivers are potentially less diverse. Consequently, compared to alluvial plains, there are fewer studies on alluvial fans and information remains scarce. As a result, progress in ecosystem conservation and restoration in these environments is limited.

This study was conducted to clarify the fish community structure in rivers flowing through small alluvial fans, where species diversity is believed to be reduced due to the natural disturbances associated with abundant sediment supply and anthropogenic riverbed dredging for flood control measures.

## Sampling methods

### Study extent

**Figure 1.**
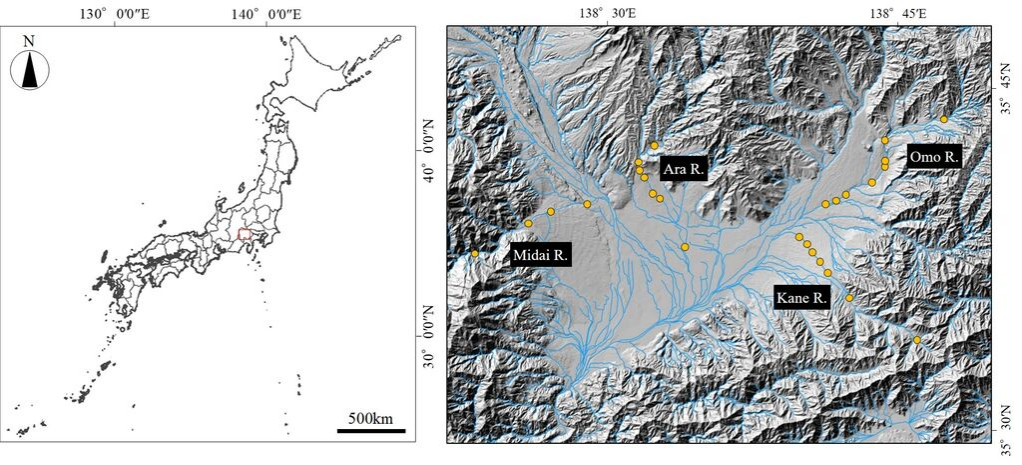
Location of the study sites. The study focused on 26 sites within the Kofu Basin.

The study area is located within the Fuji River Basin, Japan (Fig. 1). Fish surveys were conducted using an electrofisher (Smith-Root Model 12-A, Smith-Root Inc., Vancouver, WA, U.S.A.) at each study site. Fish temporarily immobilised by electric current were collected using dip nets, identified to species and subsequently released at their respective capture sites. The survey was conducted between 22 October and 29 October 2024.

### Sampling description

Fish surveys were conducted using an electrofisher (Smith-Root Model 12-A, Smith-Root Inc., Vancouver, WA, U.S.A.) at each study site. The survey length was generally set to one reach (approximately ten times the width of the river). The collected fish species were identified on-site and released immediately. Additionally, the wet weight of each species was measured at each site. In this research, we recorded 2,265 individuals which were identified on-site and in the laboratory according to Kawanabe and Mizuno (1989) and Seno (2007).

## Geographic coverage

### Description

The study area is located within the Fuji River Basin, which flows through the Fossa Magna zone, the boundary between the ichthyofaunas of south-western and north-eastern Japan (Itsukushima 2019). The study sites consist of 26 locations across four tributaries – Midai River, Ara River, Kane River and Omo River – within the Kofu Basin, each exhibiting distinct watershed characteristics. All of these rivers form alluvial fans and the study focused on locations with similar riverbed gradients. These four rivers are formed by different geological substrates: the Midai River by basalt, the Ara River by granite and rhyolite, the Kane River by diorite and the Omo River by granodiorite. Of the 26 study sites, 17 have undergone recent riverbed dredging, while nine have not.

### Coordinates

35.57553 and 35.73237 Latitude; 138.3801 and 138.7913 Longitude.

## Taxonomic coverage

### Description

The survey results revealed that a total of 15 species and one genus (only the genus could be identified) within seven families were observed, with 2,265 individuals recorded across the 26 sites. The location with the highest number of species was downstream of the Kane River, where 10 species were found. The location with the highest number of individuals was in the middle reaches of the Kane River, with 260 individuals recorded. In contrast, no fish species were captured in the upper reaches of the Midai River. The species with the highest number of individuals captured was *Rhinogobius* sp., with 818 individuals recorded across 20 sites. This was followed by *Rhynchocypris lagowskii steindachneri* Sauvage, 1883, with 450 individuals captured across 21 sites.

**Figure 2.**
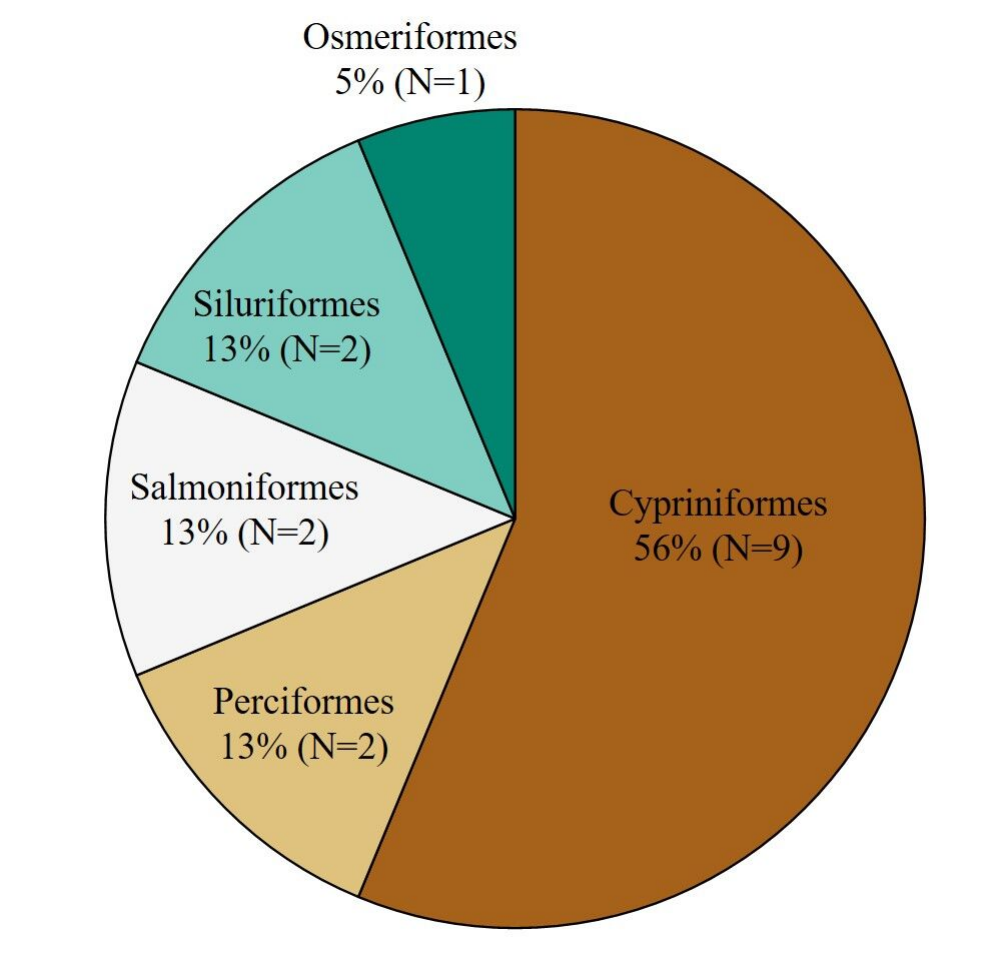
Taxonomic Coverage (by order).

**Figure 3.**
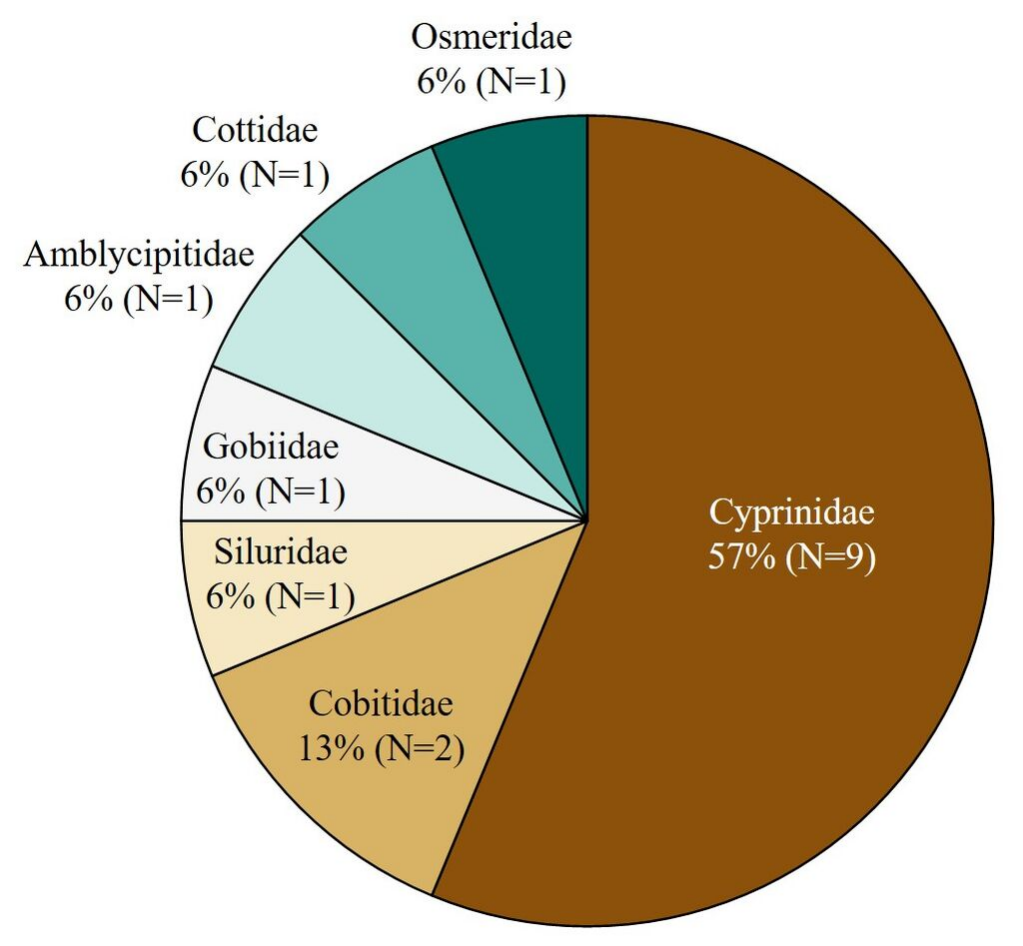
Taxonomic Coverage (by family).

The taxonomic orders observed were Cypriniformes (9 species), Perciformes (2 species), Salmoniformes (2 species), Siluriformes (2 species) and Osmeriformes (1 species) (Fig. 2). At the family level, the following families were represented: Cyprinidae (9 species), Cobitidae (2 species), Siluridae (1 species), Gobiidae (1 species), Amblycipitidae (1 species), Cottidae (1 species) and Osmeridae (1 species) (Fig. 3).

Amongst the collected fish species, those listed in the Yamanashi Prefecture Red Data Book (2018) as species at risk of extinction include *Misgurnus anguillicaudatus*, which is categorised as Data Deficient (DD), *Oncorhynchus masou ishikawae*, which is listed as a Threatened Local Population (LP) and *Cottus pollux*, which is categorised as Noteworthy Species (N). However, *Oncorhynchus masou ishikawae* has been subject to extensive transplantation (Kitanishi et al. 2017) and stocking, which facilitates hybridisation with *Oncorhynchus masou masou* (Yamazaki et al. 2005), raising the possibility that it may not be a native population. In the Ministry of the Environment's National Red Data Book (2020), in addition to the aforementioned species, *Liobagrus reini* HilGündorf, 1878 is listed as Vulnerable (VU). Furthermore, as an invasive species, *Oncorhynchus mykiss* was captured in the uppermost reaches of the Kane River, with four individuals recorded.

### Taxa included

**Table:** 

Rank	Scientific Name	
family	Salmonidae	
subspecies	*Oncorhynchus masou ishikawae* (Jordan and McGregor, 1925)	
species	*Oncorhynchus mykiss* (Walbaum, 1792)	
family	Cyprinidae	
subspecies	*Rhynchocypris lagowskii steindachneri* Sauvage, 1883	
species	*Opsariichthys platypus* (Temminck et Schlegel, 1846)	
species	*Nipponocypris temminckii* (Temminck et Schlegel, 1846)	
species	*Pseudogobio polystictus* Tominaga and Kawase, 2019	
species	*Pseudorasbora parva* (Temminck et Schlegel, 1846)	
species	*Tribolodon hakonensis* (Günther, 1877)	
species	*Gnathopogon elongatus* (Temminck et Schlegel, 1846)	
family	Cobitidae	
species	*Cobitis taenia* Linnaeus, 1758	
species	*Misgurnus anguillicaudatus* (Cantor, 1842)	
family	Siluridae	
species	*Silurus asotus* Linnaeus, 1758	
family	Gobiidae	
genus	Rhinogobius Gill, 1859	
family	Cottidae	
species	*Cottus pollux* Günther, 1873	
family	Amblycipitidae	
species	*Liobagrus reini* HilGündorf, 1878	
family	Osmeridae	
species	*Plecoglossus altivelis* (Temminck et Schlegel, 1846)	

## Temporal coverage

**Data range:** 2024-10-22 – 2024-10-29.

## Usage licence

### Usage licence

Other

### IP rights notes

This work is licensed under a Creative Commons Attribution (CC-BY 4.0) Licence.

## Data resources

### Data package title

Database of fish fauna collected from river channels on alluvial fans in the Kofu Basin, Japan

### Resource link

https://www.gbif.org/dataset/07b5664c-4dda-49e1-9f3a-c8273a7c12fb

### Alternative identifiers

https://doi.org/10.15468/madkz2

### Number of data sets

1

### Data set 1.

#### Data set name

Database of fish fauna collected from river channels on alluvial fans in the Kofu Basin, Japan

#### Download URL

https://ipt.pensoft.net/archive.do?r=database_fish_fauna_alluvial_fan_kofu_basin&v=1.2

#### Description

Surveys were conducted at 26 sites in the four rivers on alluvial fans flowing through the Kofu Basin in the central part of the Japanese archipelago. As a result of this investigation, 15 species and one genus (only the genus could be identified) within seven families were observed, with 2,265 individuals recorded across the 26 sites.

**Data set 1.** 

Column label	Column description
occurrenceID	An identifier for the Occurrence.
basisOfRecord	The specific nature of the data record.
samplingProtocol	The names of, references to, or descriptions of the methods or protocols used during an Event.
eventDate	The date-time or interval during which an Event occurred.
scientificName	The full scientific name.
scientificNameAuthorship	The authorship information for the scientificName formatted according to the conventions of the applicable nomenclaturalCode.
kingdom	The full scientific name of the kingdom in which the taxon is classified.
phylum	The full scientific name of the phylum or division in which the taxon is classified.
class	The full scientific name of the class in which the taxon is classified.
order	The full scientific name of the order in which the taxon is classified.
family	The full scientific name of the family in which the taxon is classified.
taxonRank	The taxonomic rank of the most specific name in the scientificName as it appears in the original record.
identifiedBy	A list (concatenated and separated) of names of people, groups or organisations who assigned the Taxon to the subject.
recordedBy	A list (concatenated and separated) of the globally unique identifier for the person, people, groups or organisations responsible for recording the original Occurrence.
decimalLatitude	The geographic latitude (in decimal degrees, using the spatial reference system given in geodeticDatum) of the geographic centre of a Location.
decimalLongitude	The geographic longitude (in decimal degrees, using the spatial reference system given in geodeticDatum) of the geographic centre of a Location.
coordinateUncertaintyInMetres	The horizontal distance (in metres) from the given decimalLatitude and decimalLongitude describing the smallest circle containing the whole of the Location.
geodeticDatum	The ellipsoid, geodetic datum or spatial reference system (SRS) upon which the geographic coordinates given in decimalLatitude and decimalLongitude are based.
countryCode	The standard code for the country in which the Location occurs. Recommended best practice is to use ISO 3166-1-alpha-2 country codes.
individualCount	The number of individuals represented present at the time of the Occurrence.
occurrenceStatus	A statement about the presence or absence of a Taxon at a Location.
catalogNumber	A list (concatenated and separated) of previous or alternative fully qualified catalogue numbers or other human-used identifiers for the same Occurrence, whether in the current or any other dataset or collection.
language	A language of the resource. Recommended best practice is to use a controlled vocabulary, such as RFC 4646 [RFC4646].
country	The name of the country or major administrative unit in which the Location occurs. Recommended best practice is to use a controlled vocabulary, such as the Getty Thesaurus of Geographic Names.
stateProvince	The name of the next smallest administrative region than country (state, province, canton, department, region etc.) in which the Location occurs.
municipality	The full, unabbreviated name of the next smallest administrative region than county (city, municipality etc.) in which the Location occurs. Do not use this term for a nearby named place that does not contain the actual location.
waterBody	The name of the waterbody in which the dcterms:Location occur.
modified	The four-digit year in which the Event occurred, according to the Common Era Calendar.
year	The four-digit year in which the Event occurred, according to the Common Era Calendar.
month	The ordinal month in which the Event occurred.
day	The integer day of the month on which the Event occurred.
establishmentMeans	Statement about whether a dwc:Organism has been introduced to a given place and time through the direct or indirect activity of modern humans.
dynamicProperties	A list of additional measurements, facts, characteristics or assertions about the record. Meant to provide a mechanism for structured content.

## Additional information

The species that appeared most frequently were *Plecoglossus altivelis* (Temminck et Schlegel, 1846), the genus *Rhinogobius* Gill, 1859 and the *Cobitis taenia* Linnaeus, 1758, which were widely observed. These species are the most commonly found in the gradient of the alluvial fan river channels studied (Itsukushima 2019) and they were also relatively abundant in locations affected by anthropogenic impacts, such as excavation activities.

On the other hand, *Cottus pollux* was collected in the Kane and Ara Rivers; however, its presence was not observed in the Omo River. In contrast, *Liobagrus reini* was found in the Omo River, unlike *Cottus pollux*. *Liobagrus reini* and *Cottus pollux* are distantly related species, both nocturnal benthic carnivores. It is known that they often co-exist sympatrically in the mid-stream areas of temperate rivers in Japan (Miyadi et al. 1976, Natsumeda et al. 2016). However, in this study, the rivers inhabited by *Cottus pollux* and *Liobagrus reini* were clearly separated. The difference in food resources between the two species may explain this separation. Both species primarily rely on aquatic insects as their main food source; however, *Cottus pollux* is a benthos feeder, cruising widely and preying exclusively on chironomid and trichopteran larvae. On the other hand, *Liobagrus reini* is both a drift and benthos feeder, ambushing prey and selecting ephemeropteran nymphs in addition to chironomid and trichopteran larvae (Natsumeda et al. 2016). Therefore, it is likely that the benthic faunal composition, which serves as the food resource, differs between the rivers where *Cottus pollux* and *Liobagrus reini* are found. One possible factor is the difference in the geology of the watersheds in the rivers where *Cottus pollux* and *Liobagrus reini* were observed. It is known that differences in surface geology can influence the benthic faunal composition (Shearer and Young 2011, Tashiro and Tsujimoto 2015) and these geological differences may govern the benthic faunal resources, which, in turn, could affect the presence of these predatory fish species.

Amongst the four rivers surveyed, the Midai River had significantly lower fish species richness and abundance compared to the other rivers, except at the most downstream site. The Midai River alluvial fan is a piedmont-type fan, primarily formed by the accumulation and transport of debris flows. In contrast, the other rivers traverse floor-type fans, which are basin-floor alluvial fans characterised by microtopographical features such as sandbars and natural levees formed by fluvial processes (Nakayama and Takagi 1987). Moreover, in the Midai River, engineering interventions such as channel consolidation and periodic riverbed excavation have been implemented to manage the high volume of sediment production, suggesting a greater degree of anthropogenic impact relative to the other rivers. Additionally, large-scale sediment-related disasters occurred in relatively recent periods, specifically in 1875, 1896 and 1906 (Nakayama and Takagi 1987). These events led to the extinction of many species and it is possible that the ecosystem has not yet fully recovered.

By conducting investigations on multiple rivers located in alluvial fans within the same ecological region, which are potentially inhabited by similar species, it can be suggested that the fish fauna varies depending on watershed geology, landform development processes, disaster occurrence and anthropogenic impacts. Moving forward, by continuing the research and clarifying the relationship between natural disturbances, human impacts and biodiversity, the aim is to generate findings that contribute to the conservation and restoration of alluvial fan ecosystems.
